# Substantial increase in stillbirth rate during the COVID-19 pandemic: results from a population-based study in the Indian state of Bihar

**DOI:** 10.1136/bmjgh-2023-013021

**Published:** 2023-07-25

**Authors:** Rakhi Dandona, G Anil Kumar, Md Akbar, S Siva Prasad Dora, Lalit Dandona, The ENHANCE 2020 team, Rakhi Dandona

**Affiliations:** 1 Public Health Foundation of India, New Delhi, India; 2 Institute for Health Metrics and Evaluation, University of Washington, Seattle, Washington, USA

**Keywords:** COVID-19, community-based survey, public health, maternal health, health systems

## Abstract

**Introduction:**

We report on the stillbirth rate (SBR) and associated risk factors for births during the COVID-19 pandemic, and change in SBR between prepandemic (2016) and pandemic periods in the Indian state of Bihar.

**Methods:**

Births between July 2020 and June 2021 (91.5% participation) representative of Bihar were listed. Stillbirth was defined as fetal death with gestation period of ≥7 months where the fetus did not show any sign of life. Detailed interviews were conducted for all stillbirths and neonatal deaths, and for 25% random sample of surviving live births. We estimated overall SBR, and during COVID-19 peak and non-peak periods per 1000 births. Multiple logistic regression models were run to assess risk factors for stillbirth. The change in SBR for Bihar from 2016 to 2020–2021 was estimated.

**Results:**

We identified 582 stillbirths in 30 412 births with an estimated SBR of 19.1 per 1000 births (95% CI 17.7 to 20.7); SBR was significantly higher in private facility (38.4; 95% CI 34.3 to 43.0) than in public facility (8.6; 95% CI 7.3 to 10.1) births, and for COVID-19 peak (21.2; 95% CI 19.2 to 23.4) than non-peak period (16.3; 95% CI 14.2 to 18.6) births. Pregnancies with the last pregnancy trimester during the COVID-19 peak period had 40.4% (95% CI 10.3% to 70.4%) higher SBR than those who did not. Risk factor associations for stillbirths were similar between the COVID-19 peak and non-peak periods, with gestation age of <8 months with the highest odds of stillbirth followed by referred deliveries and deliveries in private health facilities. A statistically significant increase of 24.3% and 68.9% in overall SBR and intrapartum SBR was seen between 2016 and 2020–2021, respectively.

**Conclusions:**

This study documented an increase in SBR during the COVID-19 pandemic as compared with the prepandemic period, and the varied SBR based on the intensity of the COVID-19 pandemic and by the place of delivery.

**WHAT IS ALREADY KNOWN ON THIS TOPIC**

- Significant concerns have been raised about adverse pregnancy outcomes due to COVID-19, with mixed evidence regarding stillbirths.
- Much of the literature available on change in stillbirth rate (SBR) is among women who had COVID-19 during pregnancy, with little evidence at the population level for births during the COVID-19 pandemic irrespective of COVID-19 during pregnancy.

**WHAT THIS STUDY ADDS**

- This population-based study documents birth outcomes over a time period for all pregnancies irrespective of COVID-19 infection during pregnancy, thereby allowing for an understanding of COVID-19 pandemic at the broad societal level.
- This study representative of the Indian state of Bihar documents the magnitude of increase in SBR since the start of and during the COVID-19 pandemic, and documents variations in SBR based on the intensity of the COVID-19 pandemic and by the place of delivery.
- A significant shift towards the private sector health facilities was seen between 2016 and 2020–2021, which could be due to the reluctance in using public sector facilities for delivery as many of these facilities were treating patients with COVID-19.

**HOW THIS STUDY MIGHT AFFECT RESEARCH, PRACTICE OR POLICY**

- With 4 million cumulative excess deaths due to COVID-19 estimated in India, the highest number globally, the increase in SBR documented in this study furthers the extent of possible increase in burden of disease in the country.
- The shift seen towards the private sector for delivery and the increase in stillbirth in this population emphasise the need to be vigilant in attending to the mother–fetus dyad during difficult public health emergency situations as highlighted by the COVID-19 pandemic, and to monitor the shift in care seeking and quality of labour and delivery care in public sector health facilities which may be adding to the burden of adverse outcomes.

## Introduction

Disparities in maternal and fetal outcomes are reported between the high-resource and low-resource settings during the COVID-19 pandemic.^1 2^ Significant concerns have been raised about adverse pregnancy outcomes due to COVID-19, with mixed evidence regarding stillbirths with some studies reporting an increase and some reporting no change in stillbirth rate (SBR) as a result of COVID-19 infection or lockdown.^1 3–11^ Much of the literature available on change in SBR is among women who had COVID-19 during pregnancy,^2 3 6 8 12–15^ with little evidence at the population level for births during the COVID-19 pandemic irrespective of COVID-19 during pregnancy.^7 9 10 16^ Large population-based estimates on pregnancy outcomes are missing from many countries with high number of cases of COVID-19, including from India.^9^

Despite a reduction of 53% in SBR over the past two decades, India still accounts for the largest numbers of stillbirths globally, an estimated 340 600 in 2019 translating into SBR of 13.9 (95% CI 11.4 to 17.0) per 1000 births.^17^ With the already high burden of stillbirths in India and the possibility of an increase due to COVID-19,^18^ there is a need to understand the change in SBR to monitor progress towards the India Newborn Action Plan (INAP) 2030 goal of SBR reduction.^19^ The Indian state of Bihar has one of the highest neonatal mortality rates (NMR) in the country.^20^ We have previously reported SBR at 15.4% (95% CI 13.2 to 17.9), antepartum SBR at 5.6 (95% CI 4.3 to 7.2) and intrapartum SBR at 4.5 (95% CI 3.3 to 6.1) from this state in 2016.^21^ In this paper, we report on SBR from the state for births during the COVID-19 pandemic period from July 2020 to June 2021. We assess the change in SBR between 2016 and 2020–2021, and report on the differentials in SBR and its risk factors in 2020–2021 by comparing the births during the peak of COVID-19 pandemic (months with the higher number of deaths of all ages) and those during the non-peak period.

## Methods

### Survey design

Every Newborn Health Assessment and Neonatal Care Evaluation (ENHANCE) 2020 was designed to document change in NMR in Bihar as compared with the most recent survey available for the state in the year 2016.^22^ A reduction of 23.3% in NMR in Bihar was documented over a 5-year period from NMR of 32.2 in 2011 to NMR of 24.7 per 1000 live births in 2016.^22^ To estimate the sample size for ENHANCE 2020, we assumed a similar decline in NMR between 2016 and 2020 and apportioned this reduction to 4 years at about 18%. We estimated a sample of 30 000 live births assuming a 10% refusal rate with 85% power to detect a change of 18% in NMR from 2016 to 2020.

All births that occurred between July 2020 and June 2021 among usual resident women aged 15–49 years were considered eligible for this study. Usual resident was defined as woman living in the sampled household for at least 6 months prior to the data collection. We used a multistage sampling design to obtain a representative sample of these births from all the 38 districts of Bihar. Each district of Bihar is divided into 5–27 blocks giving a total of 534 blocks in the state and we considered 50% of these blocks for the survey. We stratified the 534 blocks as those having only rural population (70.2%) and those with both rural and urban populations (29.8%), and sampled 267 blocks for the survey which included 187 (70%) blocks with only rural population and 80 (30%) blocks with both rural and urban populations. Within these 267 blocks, the secondary sampling units (SSUs) were villages in rural areas and urban frame survey blocks in urban areas as defined by the census 2011.^23^ The SSUs with <175 households were combined with an adjacent SSU, and the large rural SSUs were split into equal sized segments of 175–200 households using natural boundaries. A total of 1340 SSUs (941 rural and 399 urban) were sampled in proportion to the number of SSUs in each block using systematic random sampling.

### Data collection

Each selected SSU was mapped and all the households (a household was defined as people eating from the same kitchen) enumerated. During the enumeration, trained interviewers documented the birth outcomes between July 2020 and June 2021 among usual resident women aged 15–49 years in each household. Date of birth, sex of the baby born and whether it was a live birth or stillbirth were documented for each birth. Stillbirths were documented in enumeration by confirming that the baby did not show any sign of life (did not cry AND did not breathe AND did not move) in order to differentiate stillbirths and neonatal deaths soon after delivery. We also documented births between for women who had died during or after giving birth and those who had out-migrated during this period to ensure a robust estimation of total births in this population.

Following enumeration, all women with stillbirth and neonatal death, and 25% of women with neither selected using systematic random sampling in each SSU were considered eligible for a detailed interview. After documenting the background information including sociodemographic characteristics of the women with a birth outcome (live birth or stillbirth) in the detailed interview, questions were asked again to differentiate a stillbirth from a neonatal death that occurred soon after delivery. We documented maternal birth history and details of the pregnancy, labour, delivery, postnatal care and birth registration for the focal child of interest, and also when the baby’s last movements were felt by the mother. For stillbirths, photographs of macerated and fresh stillborn babies were shown to the respondents to facilitate documentation of baby’s appearance at birth.

Data collection was completed between August 2021 and April 2022. The questionnaire was developed in English and then translated into Hindi (local language), after which this was back-translated into English to ensure the accurate and relevant meaning and intent of the questions. Pilot testing of the questionnaire was carried out and modifications made as necessary. Interviews were captured by trained interviewers using the Computer-Assisted Personal Interview software in hand-held tablets. A total of 120 enumerators, 80 interviewers and 50 supervisors were trained by the named authors for a period of 25 days in Patna, the capital city of Bihar state. Pilot surveys were conducted to test the logistics and quality of data collection before the start of the survey. A total of 10% of the enumeration data were checked from 50% of the sampled clusters by the supervisors and MA and SSPD, which translated into 670 clusters. Similarly, a total of 20% of interviews were checked in 50% of the 1340 sampled clusters by the supervisors and MA and SSPD.

### Analysis

A stillbirth was defined as a fetal death with gestation period of ≥7 months where the baby did not show any sign of life. Data on gestation period were reported in months by the participants as is the practice in India; we assumed 1 month as 4 weeks to identify stillbirths for this analysis. We combined births en route to a health provider with the home births for this analysis. Before the analysis, data from all stillbirth interviews were reviewed to reconfirm that a stillbirth was reported based on the signs of life and gestational age. A total of 10 cases reported as stillbirth with gestation period ≤6 months were categorised as miscarriage, and seven neonatal deaths were reassigned as stillbirths and three stillbirths were reassigned as neonatal deaths.

We estimated the overall SBR in boys and girls separately, by place of delivery, place of residence and by timing of stillbirth. The stillbirths were classified as antepartum and intrapartum using the baby’s last movement felt by the mother as an indication of the time of death and the description of stillborn baby (fresh or macerated), with preference given to the baby’s movement over description as described in detail elsewhere.^21^ The change in SBR for Bihar state from 2016 to 2020–2021, and the change in the coverage of select service delivery indicators that were associated with stillbirth in this population in 2016 were estimated.^21^ There were two peaks of COVID-19 in India, between August and November 2020 and between April and June 2021, totalling to 7 months of the 12 months studied in the survey.^24^ As the proportion of stillbirths was similar between the births in the two peak periods (p=0.22; Χ^2^ test), we considered the births during these 7 months together as COVID-19 peak period births. We estimated SBR separately for births during the COVID-19 peak period and for those during the COVID-19 non-peak period. We explored the risk of stillbirths with the number of months during the last trimester of pregnancy that were during the COVID-19 peak period, and if the coverage of select service delivery indicators varied based on the number of months during the last trimester of pregnancy that were during the COVID-19 peak period. The sampling weight with design effect was applied to all the estimated SBRs to adjust for Bihar’s population, and 95% CIs are reported. Enumeration data covering all live births and stillbirths were used for all the SBR estimates.

Associations between various risk factors and stillbirth were explored, for all stillbirths and for stillbirths during or outside peak COVID-19 periods, using a hierarchical approach to build the logistic regression model,^25 26^ as reported from this population in 2016.^21^ The detailed survey data were used for this analysis. We ran five models for each analysis adjusted for place of residence and sex of the baby a priori regardless of significance, and each sequential model incorporated a group of variables considered under a particular risk factor theme from the preceding model if p value was <0.2 (value for at least one category to be <0.2 for multiple category variable).^27^ The risk factor themes included sociodemographic factors, maternal risk factors and pregnancy, labour and delivery-related factors. Based on our previous risk assessment of stillbirths,^21 28^ ‘deferred delivery’ was included as a risk factor which indicates if the woman had reported to a health provider for delivery but she was sent back by the health provider to come later for delivery. ‘Referred delivery’ in the model indicates if the woman had gone to a health provider for delivery but was referred to another health provider for delivery. Birth weight was not considered in the logistic regression as it was not available for 79.2% of the stillborn babies. We estimated the wealth index quartile to which the woman belonged to using the standard methods used in the National Family Health Survey.^29^ OR with 95% CI is presented for these models of regression results. We explored if the reasons for referred deliveries were different between the COVID-19 peak and non-peak periods. The coverage of stillbirth registration in the vital registration system is also reported. All analyses were performed using STATA V.13.1 software (StataCorp, USA).

### Ethics approval

The research conformed to the principles embodied in the Declaration of Helsinki. All participants provided written informed consent, and for those who could not read or write, the participant information sheet and consent form were explained by the trained interviewer, and a thumb impression was obtained.

## Results

### Stillbirth rate

A total of 30 412 births between July 2020 and June 2021 were identified during the enumeration from 261 124 households (91.5% participation) covering a population of 1 260 984. We identified 582 stillbirths giving an estimated SBR of 19.1 (95% CI 17.7 to 20.7) per 1000 births for the state (table 1). A statistically significant increase of 24.3% (95% CI 10.0% to 38.5%) in the overall SBR was documented for the state between 2016 and 2020–2021. The SBR for boys was higher (21.6; 95% CI 19.5 to 24.0) than for girls (16.3; 95% CI 14.4 to 18.5); and that for private facility births (38.4; 95% CI 34.3 to 43.0) and home/en route births (24.6; 95% CI 20.9 to 28.8) was significantly higher than the SBR for public facility births (8.6; 95% CI 7.3 to 10.1). The classification process for the identification of antepartum and intrapartum deaths is summarised in online supplemental figure 1. Antepartum SBR was estimated at 7.9 (95% CI 7.0 to 9.0) and intrapartum SBR as 7.6 (95% CI 6.7 to 8.6) per 1000 births in 2020–2021; and the rest (2.8%) could not be classified. The per cent increase in intrapartum SBR was 68.8% (95% CI 40.1% to 97.5%) between 2016 and 2020–2021 with no change in the antepartum SBR.

10.1136/bmjgh-2023-013021.supp1 Supplementary data

**Table 1:** Stillbirth rate (SBR) in the Indian state of Bihar, overall, by sex, place of delivery and place of residence, 2020–2021

Variables of interest	Variable categories	All births			Births during the COVID-19 peak period			Births during the COVID-19 non-peak period		
		Births (n)	Stillbirths (n)	SBR (95% CI)	Births (n)	Stillbirths (n)	SBR (95% CI)	Births (n)	Stillbirths (n)	SBR (95% CI)
Overall		30 412	582	19.1 (17.6 to 20.7)	17 798	377	21.2 (19.2 to 23.4)	12 614	205	16.3 (14.2 to 18.6)
Sex of the baby	Boy	15 959	345	21.6 (19.5 to 24.0)	9252	214	23.1 (20.3 to 26.4)	6707	131	19.5 (16.5 to 23.1)
	Girl	14 452	236	16.3 (14.4 to 18.5)	8545	162	19.0 (16.3 to 22.1)	5907	74	12.5 (10.0 to 25.7)
Place of delivery	Public facility	16 898	145	8.6 (7.3 to 10.1)	9737	92	9.4 (7.7 to 11.6)	7161	53	7.4 (5.7 to 9.7)
	Private facility	7522	289	38.4 (34.3 to 43.0)	4398	185	42.1 (36.5 to 48.4)	3124	104	33.3 (27.5 to 40.2)
	Home	5983	147	24.6 (20.0 to 28.8)	3661	99	27.0 (22.3 to 32.8)	2322	48	20.7 (15.6 to 27.3)
Place of residence	Rural	24 594	471	19.2 (17.5 to 20.9)	14 338	299	20.9 (18.6 to 23.3)	10 256	172	16.8 (14.5 to 19.4)
	Urban	5818	111	19.1 (15.9 to 22.9)	3460	78	22.5 (18.1 to 28.1)	2358	33	14.0 (10.0 to 19.6)
Timing of stillbirth	Antepartum	30 412	241	7.9 (7.0 to 9.0)	17 798	159	8.9 (7.7 to 10.4)	12 614	82	6.5 (5.2 to 8.1)
	Intrapartum	30 412	231	7.6 (6.7 to 8.6)	17 798	149	8.4 (7.1 to 9.8)	12 614	82	6.5 (5.2 to 8.1)
	Unclassified	30 412	110	3.6 (3.0 to 4.4)	17 798	69	3.9 (3.1 to 4.9)	12 614	41	3.3 (2.4 to 4.4)

CI, confidence interval.

#### SBR by COVID-19 peak and non-peak periods

Of the 30 412 births, 17 798 (58.5%) were born during the COVID-19 peak period including 377 (2.1%) stillbirths, and the rest during the COVID-19 non-peak period including 205 (1.6%) stillbirths. The SBR per 1000 births among COVID-19 peak period births was significantly higher (21.2; 95% CI 19.2 to 23.4) as compared with the COVID-19 non-peak period births (16.3; 95% CI 14.2 to 18.6) as shown in table 1. The per cent change in SBR between COVID-19 peak and non-peak periods was significant for private facility births (26.4%; 95% CI 0.5% to 52.4%), rural births (24.4%; 95% CI 4.1% to 44.7%) and urban births (60.7%; 95% CI 11.8% to 109.6%), but was not statistically significant for public facility births (27%; 95% CI −10.3% to 64.3%) and home births (30.4%; 95% CI −7.3% to 68.2%).

The antepartum and intrapartum SBRs were estimated at 37% and 29% higher during the COVID-19 peak period as compared with the non-peak period, respectively, but the difference was not statistically significant (table 1). The distribution of stillbirths by gestation period for antepartum and intrapartum stillbirths was similar between COVID-19 peak and non-peak periods (online supplemental table 1). The sex distribution among antepartum stillbirth was similar between boys and girls during the COVID-19 peak period; however, antepartum stillbirths were comparatively higher in boys (63.4%) than girls (36.6%) during the COVID-19 non-peak period. Among intrapartum stillbirths, the proportion of boys (63.5% and 61%) was higher than the girls (36.5% and 39%) in both COVID-19 peak and non-peak periods.

### Risk factor analysis

Of the 8853 eligible births for detailed interview, 7270 mothers participated (82.1% participation), including 501 mothers of stillbirths (86.1% participation) and 6769 mothers of live births (81.8% participation). Data on COVID-19 infection during pregnancy were available for 6531 births (89.6%), of whom only nine (0.14%) reported having this infection during pregnancy. None of the nine pregnancies with COVID-19 infection resulted in a stillbirth. For the 4263 births in the survey during the COVID-19 peak period, only 30% women reported having tested for COVID-19 at the time of delivery (35% in public sector and 41% in private sector).

The change in select service indicators for all births and stillbirths between 2016 and 2020–2021 is shown in online supplemental table 2. A statistically significant per cent increase was documented over this period for all the indicators for all births; however, a statistically significant change was not seen for mothers being informed of baby not growing adequately inside the womb, referred delivery, caesarean section (C-section) and push/forceful pull used during delivery by healthcare providers when considering only stillbirths.

**Figure 1:**
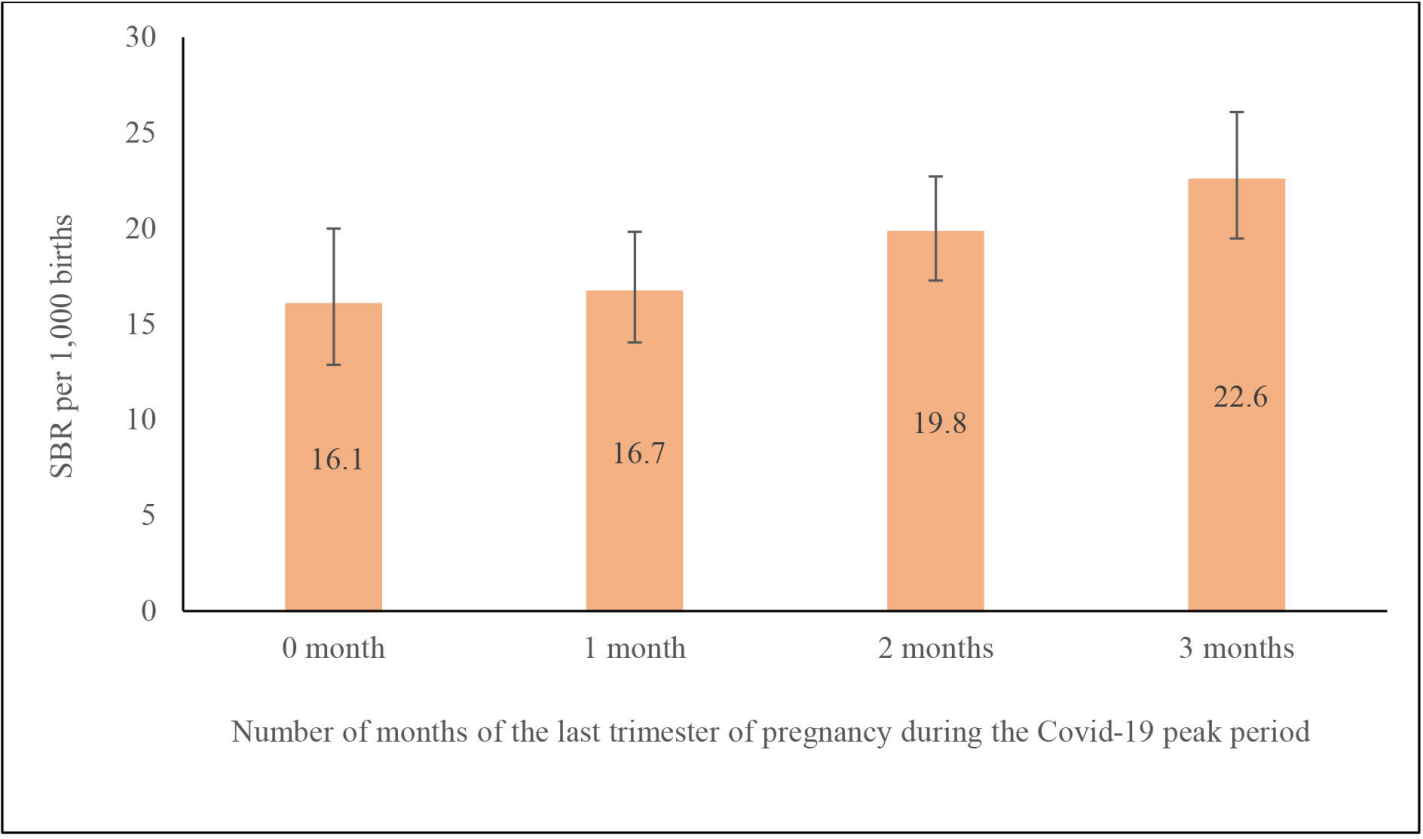
Stillbirth rate (SBR) on the number of months in the last trimester of pregnancy that were in the COVID-19 peak period, 2020–2021. Bars denote 95% CI.

The distribution of all births and stillbirths during COVID-19 and COVID-19 non-peak periods by select risk factors is documented in online supplemental table 3. The proportion of stillbirths was higher in women who had all of their last trimester of pregnancy during the peak COVID-19 months as compared with those who did not have the last trimester during this period (p=0.019; online supplemental figure 2). The SBR for pregnancies with all the 3 months of the last trimester of pregnancy during the peak COVID-19 months was 22.6 (95% CI 19.5 to 26.1), which was 40.4% (95% CI 10.3% to 70.4%) higher than the SBR for pregnancies with no month of the last trimester of pregnancy during this period (figure 1).

There was no difference in the distribution of number of antenatal care (ANC) visits for the women by the number of months in the last trimester that were during the COVID-19 peak period (online supplemental figure 3). There was also no statistically significant difference by the gestation period, deferred and referred deliveries and place of delivery by the number of months in the last trimester that were during the COVID-19 peak period (online supplemental table 4).

#### Logistic regression results

The sequential logistic regression models 1–4 for all births, and those in COVID-19 peak and non-peak periods are shown in online supplemental tables 5–7, and the final model 5 is shown in table 2. Considering all births irrespective of COVID-19, gestation age of 7 months (OR 7.77; 95% CI 5.53 to 10.94) had the highest odds for stillbirth, followed by for those reporting push/pull (manual fundal pressure/forceful pulling of the baby) during the delivery by health provider (OR 5.82; 95% CI 4.44 to 7.64) and referred deliveries (OR 3.22; 95% CI 2.44 to 4.26). In addition, boy fetus, history of stillbirth, deliveries other than those at public sector facilities, breech presentation of the baby and pregnancies with 2 or more months of the last trimester also had significantly higher odds of stillbirth (table 2).

**Table 2:** Results of the final sequential multiple logistic regression model (model 5) for association of stillbirth with sociodemographic, maternal, pregnancy, labour and delivery-related risk factors in the Indian state of Bihar for all births, for births during the COVID-19 peak period and for births during the COVID-19 non-peak period

Risk factor	Risk factor categories	OR for stillbirth (95% CI)		
		All births	COVID-19 peak period births	COVID-19 non-peak period births
Place of residence	Rural	0.91 (0.70 to 1.17)	0.98 (0.71 to 1.34)	0.85 (0.53 to 1.37)
	Urban	1.00	1.00	1.00
Sex of the baby	Boy	**1.26 (1.04 to 3.54)**	1.19 (0.93 to 1.53)	**1.46 (1.04 to 2.05)**
	Girl		1.00	1.00
Wealth index quartile	I (lowest)			1.36 (0.80 to 2.33)
	II			**1.84 (1.12 to 3.04)**
	III			1.38 (0.83 to 2.30)
	IV (highest)			1.00
Maternal age (years)	15–19	1.03 (0.61 to 1.74)		
	20–24	0.80 (0.53 to 1.21)		
	25–29	0.83 (0.54 to 1.26)		
	30–34	1.01 (0.64 to 1.59)		
	≥35	1.00		
Solid cooking fuel use	Yes			
	No			
First-born	Yes		1.26 (0.95 to 1.67)	
	No		1.00	
History of stillbirth	Yes	**2.00 (1.42 to 2.81)**	**2.29 (1.48 to 3.53)**	**1.94 (1.11 to 3.38)**
	No	1.00	1.00	1.00
History of miscarriage	Yes			
	No			
Maternal antenatal care visit during pregnancy	No	1.48 (0.97 to 2.26)		**2.83 (1.55 to 5.14)**
	Yes	1.00		1.00
Mother received 2 tetanus toxoid injections during pregnancy.	Yes	1.00		
	No	1.21 (0.98 to 1.50)		
Mother consumed iron folic acid tablets during pregnancy.	Yes	1.00	1.00	
	No	1.25 (0.98 to 1.60)	**1.52 (1.15 to 2.00)**	
Pregnancy with multiple fetuses	Yes			
	No			
Maternal hypertension in the last trimester of pregnancy	Yes			
	No			
Mother had malaria in the last trimester of pregnancy.	Yes			
	No			
Mother was diagnosed with syphilis during this pregnancy.	Yes			
	No			
Mother had fever in the last 3 months of pregnancy.	Yes	1.08 (0.76 to 1.54)	**1.53 (1.03 to 2.28)**	**0.43 (0.19 to 0.97)**
	No	1.00	1.00	1.00
Mother had convulsions in the last 3 months of pregnancy.	Yes			1.43 (0.92 to 2.22)
	No			1.00
Mother was informed baby was not growing adequately inside the womb.	Yes			
	No			
Number of months in the last trimester of pregnancy during the COVID-19 peak period	0	1.00		
	1	1.15 (0.81 to 1.63)	1.00	
	2	**1.48 (1.07 to 2.04)**	**1.47 (1.04 to 2.08)**	
	3	**1.66 (1.19 to 2.30)**	**1.45 (1.05 to 2.00)**	
Gestation period (months)	7	**7.77 (5.53 to 10.94)**	**8.08 (5.28 to 12.35)**	**8.47 (4.73 to 15.18)**
	>7 to 8	**1.60 (1.29 to 1.98)**	**1.55 (1.19 to 2.04)**	**1.72 (1.20 to 2.45)**
	>8	1.00	1.00	1.00
Deferred delivery	Yes			
	No			
Delivery was referred to another healthcare provider.	Yes	**3.22 (2.44 to 4.26)**	**3.03 (2.11 to 4.35)**	**3.10 (1.98 to 4.85)**
	No	1.00	1.00	1.00
Spontaneous labour	Yes	**0.66 (0.49 to 0.88)**	**0.54 (0.38 to 0.77)**	
	No	1.00	1.00	1.00
Place of delivery	Public facility	1.00	1.00	1.00
	Private facility	**2.66 (2.01 to 3.52)**	**2.42 (1.69 to 3.45)**	**3.46 (2.18 to 5.48)**
	Home	**2.25 (1.68 to 2.96)**	**2.65 (1.86 to 3.76)**	**2.03 (1.26 to 3.28)**
	En route to the facility	**2.64 (1.31 to 5.27)**	**3.91 (1.73 to 8.85)**	1.29 (0.29 to 5.77)
Vaginal delivery	Yes	1.22 (0.89 to 1.68)	1.17 (0.77 to 1.76)	1.38 (0.83 to 2.27)
	No			
Push/forceful pull was done during delivery by the health provider.	Yes	**5.82 (4.44 to 7.64)**	**7.31 (5.21 to 10.28)**	**4.08 (2.56 to 6.50)**
	No	1.00	1.00	1.00
Entangled cord around the baby’s neck	Yes	0.55 (0.25 to 1.20)	0.65 (0.25 to 1.67)	0.41 (0.09 to 1.89)
	No	1.00	1.00	1.00
Breech presentation of the baby	Yes	**2.05 (1.49 to 2.82)**	**1.67 (1.10 to 2.54)**	**2.84 (1.71 to 4.73)**
	No	1.00	1.00	1.00

Statistically significant ORs are shown in bold.

Much of the risk factor associations for stillbirth were similar between the COVID-19 peak and non-peak periods (online supplemental tables 6 and 7 and table 2), including the higher odds for births with gestation age of 7 months (OR 8.08; 95% CI 5.28 to 12.35 for COVID-19 peak period and OR 8.47; 95% CI 4.73 to 15.18 for non-peak period), referred deliveries, push/forceful pull during the delivery by the health provider, breech position of the baby, births in private facilities and home and maternal history of stillbirth (table 2). The risk factors significant only for COVID-19 peak period were mothers not having consumed iron folic acid tablets during pregnancy, pregnancies with 2 or more months of the last trimester and spontaneous labour (table 2). Interestingly, no ANC visit during pregnancy (OR 2.83; 95% CI 1.55 to 5.14) and belonging to wealth index quartile II (OR 1.84; 95% CI 1.12 to 3.04) were significant risk factors during the COVID-19 non-peak period but not during the COVID-19 peak period.

Among the 124 stillbirths that were referred deliveries, 67.3% of them were referred from the public sector to private sector health facility, and 14.6% were referred between the private sector facilities. The reasons for referral (not mutually exclusive) included complicated delivery (74.2%), for C-section (24.2%), provider refused to deliver a dead baby (19.4%), bleeding during labour (12.4%) and refusal of treatment by health provider (10.5%). These reasons for referral were similar during COVID-19 peak and non-peak periods (data not shown).

### Stillbirth registration

Stillbirth registration was reported only for 10 (2%) of the 501 stillbirths who participated in the detailed survey, and the certificate was shown for eight of them. The reasons for not registering stillbirths (not mutually exclusive) included no need to register a born dead baby (87.4%), was not aware of such a registration (12.6%) and did not think about registration (10.2%).

## Discussion

In our understanding, this is the first population-based study from India highlighting the magnitude of increase in SBR during the COVID-19 pandemic. In addition to validating the concerns of an increase in SBR during the pandemic versus the prepandemic period, the study has also documented variations in SBR based on the intensity of the COVID-19 pandemic and by the place of delivery, and varied risk factors based on the intensity of the COVID-19 pandemic.

With India already having the highest numbers of excess deaths due to COVID-19 globally,^30^ our finding of an increase in the SBR in Bihar during the pandemic suggests there is an even larger COVID-19 mortality burden in India than previously understood. The overall SBR in the state increased by 24%, whereas intrapartum SBR increased by nearly 70% between 2016 and 2020–2021. The increase in SBR as a result of COVID-19 reported from other parts of the world was attributed to reduced antenatal surveillance, a reluctance to access in-hospital care due to increased stress and anxiety and missed appointments due to rapid changes in maternity services during the pandemic.^5 10 31–34^ We did not find a decrease in the coverage of services but documented a significant shift towards the private sector health facilities over this period, which could be due to the reluctance in using public sector facilities for delivery as many of these facilities were treating patients with COVID-19.^35^ This shift possibly countered the access-related issues for maternal and newborn services in this population,^36^ a phenomenon also documented elsewhere.^37^ On the other hand, this shift also possibly resulted in an increase in stillbirths as the newborn survival in India is known to be influenced by the place of delivery, with the private sector performing poorly for early neonatal deaths in comparison to the public sector including in Bihar.^38^ We have also previously reported higher SBRs and NMRs in the private sector as compared with the public sector facilities from the state.^21 22^ Poor quality of healthcare is reported to be a major driver of excess mortality across a variety of disease conditions globally, including for newborn deaths.^39 40^ One could hypothesise the significant increase in the intrapartum SBR in this population to be a result of poor quality of intrapartum services in the private sector, which also aligns with the higher estimates of early neonatal mortality in these facilities. The private sector health facilities in the state are not a homogenous group and range from tertiary care hospitals to nursing homes with varied levels of infrastructure, capacity and skills.^36^ On the other hand, it is also important to interpret the higher SBR in private sector facilities within the context of referral for delivery in this population. Most deliveries that were referred were reported to be complicated deliveries, and majority of this referral was from the public sector to the private sector facilities. It is well known that the private sector in India provides most of the emergency obstetric care and also serves as a referral facility for public sector for complicated deliveries.^41 42^ This is despite the government of India’s ambitious initiative, the labour room and quality improvement initiative (*LaQshya*), launched in 2017 to improve quality of labour and delivery care in the public sector facilities.^43^ The INAP also has quality of care as one of the six guiding principles, and care during labour and childbirth is one of the strategic intervention packages.^19^ And yet, push/pull by the healthcare provider and breech position of the fetus continue to be important risk factors for stillbirth in this population,^21 28^ highlighting that the skills of staff providing emergency obstetric care continue to remain poor.^44–48^ Furthermore, the shift seen towards the private sector for delivery and the increase in stillbirth in this population in 2020–2021 emphasise the need to be vigilant in attending to the mother–fetus dyad during difficult public health emergency situations as highlighted by the COVID-19 pandemic, and emphasise the need to monitor the shift in care seeking by the community and the need to attend to the continued poor capacity of the public sector to provide care for complicated deliveries, which may be adding to the burden of adverse outcomes. Engagement with the private sector and understanding the adverse outcomes by the type of private sector is urgently needed to address adverse pregnancy outcomes.^49 50^

Overall, the risk factors associated with stillbirths in 2020–2021 were similar to those as identified in this population in 2016, with the strongest risk factor being a short gestational age (7 months) as shown by large OR^21^; however, some differences were noted in risk factors between the births in COVID-19 peak and non-peak periods in 2020–2021. The finding of nearly 50% higher odds for SBR in pregnancies with 2 or more months of the last trimester of pregnancy during the peak COVID-19 months is quite intriguing. It is important to note that some births in the second peak would also have had pregnancies starting during the first COVID-19 peak, but it is difficult for us to comment on such complexities based on our study. Furthermore, the reporting of COVID-19 infection during pregnancy in our population was negligible and the coverage of COVID-19 testing during delivery was also poor for the COVID-19 peak period births. The COVID-19 peak period was when India had most COVID-19-related deaths; a period during which there was no lockdown but the health system had almost crashed across the country.^51–53^ It is likely that increase in stillbirths was a result of poorly prepared and inadequately equipped health system, in particular for intrapartum care^54 55^; however, more detailed and nuanced exploration is needed to understand the possible pathways for this finding in order to be better prepared to address adverse pregnancy outcomes in future public health emergencies.

A much higher proportion of boys than girls were documented in antepartum during the COVID-19 non-peak period in our study. A 10% higher risk of stillbirth in male fetus has been reported from a meta-analysis of more than 30 million births globally but it did not comment if the sex was linked to the timing of stillbirth.^56^ We cannot comment on the higher proportion of antepartum stillbirths in boys, but an understanding of why males are at higher risk is a research priority that could potentially lead to sex-specific approaches to the management of high-risk pregnancies.^56^

Registration of stillbirths was negligible with only 2% registered, despite the stillbirth registration being mandatory in India.^57^ The missed opportunity to improve the documentation of stillbirths is significant with 75% of these babies born in health facilities, where the health facility in charge or the doctor is required to report the stillbirths to the vital registration system.

There are several strengths of this study. Many of the published pooled analyses reporting on population-based estimates on pregnancy outcomes as a result of COVID-19 include only a small number of studies from India, a country with very high COVID-19 case numbers and related mortality.^30^ These large-scale state-wide representative data on all births allowed not only for a detailed understanding of epidemiology of stillbirths by place of delivery and by type, but additionally having baseline of SBR and risk factors in the same population previously allowed for understanding of the type and extent of changes between prepandemic and pandemic periods. Importantly, this was a population-based study of pregnancy outcomes over a time period and did not evaluate the difference in outcomes between those women who tested positive for COVID-19 or were symptomatic and those women who tested negative. We were interested in understanding whether there was an increase in stillbirth during the pandemic and if so, whether this increase was associated with a decrease in services. We also generated robust SBR estimates by documenting all in/out-migration among the women of reproductive age who had a pregnancy outcome in the period of interest to provide an appropriate denominator for the stillbirth estimation. We strengthened the numerator for SBR estimates by confirming stillbirth at three points in time from enumeration through the analysis by confirming signs of life, which is different from the Demographic and Health Surveys (DHS) surveys which do not confirm signs of life from the respondent when documenting a stillbirth.^29^ We believe that the study findings are potentially generalisable to other less developed states of India, and the context could be different in the more developed states of the country given the stronger health systems in the latter.^20^

There are some limitations also to the study findings. The gestational age was captured in months instead of weeks as the pregnancy length in India is reported in months. The last menstrual period forms the basis for most gestational age estimates and is considered a reliable estimate for measuring gestational age in both developing and developed country settings.^58 59^ To classify stillbirths as antepartum or intrapartum, a previously used cut-off of within 8 hours since the last felt baby’s movement was considered,^21 28 60 61^ and we preferred the baby’s movement over the description of stillborn baby because appearance is reported to be a less accurate proxy for death-to-delivery interval.^62–64^

## Conclusion

The COVID-19 pandemic has potentially derailed on-track efforts to achieve the Sustainable Development Goals by 2030, especially for maternal and neonatal survival including stillbirth prevention, and urgent efforts are needed to build stronger health systems after the pandemic.^65^ In addition to documenting the extent of increase in stillbirths as a result of the pandemic, our study findings highlight the opportunities and challenges of private sector delivery of maternal and newborn care as a result of the pandemic, and the need to accelerate improvements in quality of labour and delivery care in the public sector facilities.^66^ It also adds to the call on strengthening death registration systems around the world, which is crucial to global public health strategy and necessary for improved monitoring of this pandemic and future pandemics.^30^

## Data Availability

All data relevant to the study are included in the article or uploaded as supplementary information.
