# Supplementary material for: Substantial increase in stillbirth rate during the COVID-19 pandemic: results from a population-based study in the Indian state of Bihar
Source: BMJ Glob Health. 2023 Jul 25;8(7):e013021. doi: 10.1136/bmjgh-2023-013021 (PMC10373740; doi:10.1136/bmjgh-2023-013021)

Supplementary Table 1. Distribution of gestation age by timing of stillbirths.

	Antepartum stillbirth[a]		Intrapartum stillbirth[b]	
**Gestational age**	**Covid-19 peak period N=159 (% of N)**	**Covid-19 non-peak period N=82 (% of N)**	**Covid-19 peak period N=149 (% of N)**	**Covid-19 non-peak period N=82 (% of N)**
7 months	22 (13.8)	10 (12.2)	28 (18.8)	12 (14.6)
8 months	49 (30.8)	32 (39.0)	55 (36.9)	30 (36.6)
>8 months	88 (55.4)	40 (48.8)	66 (44.3)	40 (48.8)

a p=0.442; chi square test
b p=0.683; chi square test

Supplementary Table 2. Distribution of select service delivery indicators for all births and stillbirths and percent change between 2016[18] and 2020-21.

	All births			Stillbirths		
**Service delivery indicators**	**2016** *N* = 20,152 (% of N)	**2020-21** *N* = 7,270 (% of N)	**Percent change from 2016 to 2020-21 (95% confidence interval)**	**2016** *N* = 275 (% of N)	**2020-21** *N* = 500 (% of N)	**Percent change from 2016 to 2020-21 (95% confidence interval)**
At least one antenatal care visit during pregnancy[a]	16,363 (81.2)	6,929 (95.3)	17.4 (16.5 to 18.3)	212 (78.2)	456 (91.2)	16.6 (9.6 to 23.7)
Four or more antenatal care visit during pregnancy[a]	2,446 (12.1)	2,548 (35.1)	190.1 (180.3 to 199.9)	50 (18.5)	172 (34.4)	85.9 (52.3 to 119.6)
Mother received two Tetanus injections during pregnancy[b]	16,295 (81.1)	5,037 (69.3)	-14.5 (-16.0 to -13.1)	204 (74.2)	324 (64.8)	-12.7 (-21.6 to -3.7)
Mother consumed Iron Folic Acid tablets during pregnancy[c]	8,091 (40.4)	5,609 (77.2)	91.1 (88.2 to 94.0)	84 (30.8)	348 (69.6)	126.0 (103.9 to 148.1)
Mother was informed that the baby was not growing adequately inside the womb[d]	703 (3.5)	885 (12.2)	248.6 (225.9 to 271.3)	24 (8.8)	62 (12.4)	40.9 (-9.4 to 91.2)
Delivery was deferred[e]	175 (0.9)	623 (8.6)	855.6 (782.5 to 928.6)	15 (5.5)	50 (10.0)	81.8 (13.3 to 150.3)
Mother was referred for delivery to another health care provider[f]	827 (5.9)	602 (8.3)	40.7 (28.6 to 52.8)	46 (27.5)	124 (24.8)	-9.8 (-37.9 to 18.3)
Birth in gestation age of 7 month (28-31 weeks)	179 (0.9)	223 (3.1)	244.4 (197.9 to 291.0)	39 (14.2)	81 (16.2)	14.1 (-22.8 to 51.0)
Public facility birth	10,712 (53.2)	3,707 (51.0)	-4.1 (-6.7 to -1.6)	89 (33.0)	128 (25.6)	-22.4 (-43.0 to -1.9)
Private facility birth	3,394 (16.9)	1,873 (25.8)	52.7 (46.0 to 59.4)	80 (29.6)	243 (48.6)	64.2 (40.6 to 87.8)
Home birth	6,023 (29.9)	1,689 (23.2)	-22.4 (-26.3 to -18.5)	101 (37.4)	129 (25.8)	-31.0 (-49.5 to -12.5)
C-section delivery[g]	2,028 (10.1)	940 (13.0)	28.7 (20.0 to 37.4)	37 (13.9)	93 (18.7)	34.5 (-4.2 to 73.3)
Push/forceful pull used during delivery by health care provider[h]	1,025 (5.2)	527 (7.3)	40.4 (27.5 to 53.3)	60 (22.8)	126 (25.2)	10.5 (-17.3 to 38.3)
Birth weight measured[i]	15,244 (76.4)	5,869 (80.8)	5.8 (4.3 to 7.2)	40 (15.5)	134 (26.8)	72.9 (35.0 to 110.8)

[a]Data missing for 5 births and 4 stillbirths in 2016 and 1 birth and 1 stillbirth in 2020-21; [b]Data missing for 51 births in 2016 and 1 birth and 1 stillbirth in 2020-21; [c]Data missing for 120 births and 2 stillbirths in 2016 and 1 birth and 1 stillbirth in2020-21; [d]Data missing for 240 births and 1 stillbirth in 2016 and 2 births and 2 stillbirths in 2020-21; [e]Data missing for 203 births and 1 stillbirth in 2016 and 2 births and 1 stillbirth 2020-21; [f]Data missing for 4 births and 2 stillbirths (data shown only for women who delivered in a health facility) in 2016 and 2 births and 1 stillbirth in 2020-21; [g]Data missing for 28 births and 9 stillbirths in 2016 and 2 births and 3 stillbirths in 2020-21; [h]Data missing for 391 births and 12 stillbirths in 2016 and 2 births and 1 stillbirth in 2020-21; [i]Data missing for 204 births and 16 stillbirths in 2016 and 2 births and 1 stillbirth in 2020-21.

Supplementary Table 3. Distribution of select risk factors for births and stillbirths between July 2020 and June 2021 by births in Covid-19 peak period and non-peak period.

Variables of interest		Births irrespective of Covid-19 peak period		Births in Covid-19 peak period		Births in Covid-19 non-peak period	
		All births	Stillbirths	All births	Stillbirths	All births	Stillbirths
		N = 7,270 (% of N)	*N* = 501 (% of births)	*N* = 4,263 (% of N)	*N* = 326 (% of births)	*N* = 3,007 (% of N)	*N* = 175 (% of births)
**Place of residence**	Rural	5,963 (82.0)	409 (6.9)	3,458 (81.1)	260 (7.5)	2,505 (83.3)	149 (6.0)
	Urban	1,307 (18.0)	92 (7.0)	805 (18.9)	66 (8.2)	502 (16.7	26 (5.2)
**Sex of baby**	Boy	3,087 (52.4)	296 (7.8)	2,225 (52.2)	186 (8.4)	1,582 (52.6)	110 (7.0)
	Girl	3,463 (47.6)	205 (5.9)	2,038 (47.8)	140 (6.9)	1,425 (47.4)	65 (4.6)
**Wealth index quartile**[a]	I (lowest)	1,892 (26.1)	136 (7.2)	1,144 (26.9)	92 (8.0)	748 (24.9)	44 (5.9)
	II	1,865 (25.7)	134 (7.2)	1,041 (24.4)	77 (7.4)	824 (27.4)	57 (6.9)
	III	1,807 (24.9)	112 (6.2)	1,051 (24.7)	70 (6.7)	756 (25.2)	42 (5.6)
	IV (highest)	1.700 (23.4)	118 (6.9)	1,024 (24.0)	86 (8.4)	676 (22.5)	24 (4.7)
**Maternal age in years**	15-19	488 (6.7)	42 (8.6)	288 (6.8)	30 (10.4)	200 (6.6)	12 (6.0)
	20-24	3,197 (44.0)	211 (6.6)	1,897 (44.5)	142 (7.5)	1,300 (43.2)	69 (5.3)
	25-29	2,258 (31.1)	142 (6.3)	1,320 (31.0)	92 (7.0)	938 (31.2)	50 (5.3)
	30-34	905 (12.5)	70 (7.7)	514 (12.1)	41 (8.0)	391 (13.0)	29 (7.4)
	35+	422 (5.8)	36 (8.5)	244 (5.7)	21 (8.6)	178 (5.9)	15 (8.4)
**First born**[b]	No	5,422 (74.6)	359 (6.6)	3,136 (73.6)	220 (7.0)	2,286 (76.0)	139 (6.1)
	Yes	1,846 (25.4)	141 (7.6)	1,125 (26.4)	105 (9.3)	721 (24.0)	36 (5.0)
**Previous history of stillbirth**	No	6,871 (94.5)	447 (6.5)	4,030 (94.5)	291 (7.2)	2,841 (94.5)	156 (5.5)
	Yes	399 (5.5)	54 (13.5)	233 (5.5)	35 (15.0)	166 (5.5)	19 (11.5)
**Previous history of miscarriage**	No	6,273 (86.3)	423 (6.7)	3,685 (86.4)	271 (7.4)	2,588 (86.1)	152 (5.9)
	Yes	997 (13.7)	78 (7.8)	578 (13.6)	55 (9.5)	419 (13.9)	23 (5.5)
**At least one ANC visit during pregnancy**[c]	No	340 (4.7)	44 (12.9)	217 (5.1)	25 (11.5)	123 (4.1)	19 (15.5)
	Yes	6,929 (95.3)	456 (6.6)	4,045 (94.9)	300 (7.4)	2,884 (95.9(	156 (5.4)

Variables of interest		Births irrespective of Covid-19 peak period		Births in Covid-19 peak period		Births in Covid-19 non-peak period	
		All births	Stillbirths	All births	Stillbirths	All births	Stillbirths
		N = 7,270 (% of N)	*N* = 501 (% of births)	*N* = 4,263 (% of N)	*N* = 326 (% of births)	*N* = 3,007 (% of N)	*N* = 175 (% of births)
**Mother received 2 Tetanus injections during pregnancy**[c]	No/Don't know	2,232 (30.7)	176 (7.9)	1,331 (31.2)	116 (8.7)	901 (30.0)	60 (6.7)
	Yes	5,037 (69.3)	324 (6.4)	2,931 (68.8)	209 (7.1)	2,106 (70.0)	115 (5.5)
**Mother consumed iron folic acid tablets during pregnancy**[c]	No/Don't know	1,661 (22.9)	153 (9.2)	949 (22.3)	99 (10.5)	712 (23.7)	54 (7.6)
	Yes	5,609 (77.1)	348 (6.2)	3,314 (77.7)	227 (6.9)	2,295 (76.3)	121 (5.3)
**Pregnancy with multiple foetuses**	Single	7,077 (97.4)	472 (6.7)	4,138 (97.1)	306 (7.4)	2,939 (97.7)	166 (5.7)
	Multiple	193 (2.6)	29 (15.0)	125 (2.9)	20 (16.0)	68 (2.3)	9 (13.2)
**Maternal hypertension in last trimester of pregnancy**[b]	No/Don't know	7,048 (97.0)	478 (6.8)	4,138 (97.1)	310(7.5)	2,910 (96.8)	168 (5.8)
	Yes	220 (3.0)	22 (10.0)	124 (2.9)	15 (12.1)	96 (3.2)	7 (7.3)
**Mother had malaria in last trimester of pregnancy**[b]	No/Don't know	7,231 (99.5)	497 (6.9)	4,241 (99.5)	324 (7.6)	2,990 (99.5)	173 (5.6)
	Yes	37 (0.5)	3 (8.1)	21 (0.5)	1 (4.8)	16 (0.5)	2 (12.5)
**Mother diagnosed with syphilis during pregnancy**[b]	No/Don't know	6,990 (96.2)	484 (6.9)	4,100 (96.2)	314 (7.7)	2,890 (96.1)	170 (5.9)
	Yes	278 (3.8)	16 (5.8)	152 (3.8)	11 (6.8)	116 (3.9)	5 (4.3)
**Mother had fever in last 3 months of pregnancy**[b]	No/Don't know	6,723 (92.5)	452 (6.7)	3,924 (92.1)	285 (7.3)	2,799 (93.1)	167 (6.0)
	Yes	545 (7.5)	48 (8.8)	338 (7.9)	40 (11.8)	207 (6.9)	8 (3.9)
**Mother had convulsions in last 3 months of pregnancy**[b]	No/Don't know	6,350 (87.4)	431 (6.8)	3,729 (87.5)	287 (7.7)	2,621 (87.2)	144 (5.5)
	Yes	918 (12.6)	69 (7.5)	533 (12.5)	38 (7.1)	385 (12.8)	31 (8.0)
**Mother was informed that the baby was not growing adequately inside the womb**[b]	No	6,379 (87.8)	437 (6.9)	3,730 (87.6)	283 (7.6)	2,649 (88.2)	154 (5.8)
	Yes	885 (12.2)	62 (7.0)	530 (12.4)	41 (7.7)	355 (11.82)	21 (5.9)
**Gestation age at the time of delivery**	7 months	223 (3.1)	81 (36.3)	141 (3.3)	55 (39.0)	82 (2.7)	26 (31.7)
	8 months	2,293 (31.5)	177 (7.7)	1,339 (31.4)	10 (8.2)	954 (31,7)	67 (7.0)
	>8 months	4,754 (65.4)	243 (5.1)	2,783 (65.3)	161 (5.8)	1,971 (65.6)	82 (4.2)
**Delivery was deferred**[b]	No	6,645 (91.4)	450 (6.8)	3,892 (91.3)	291 (7.5)	2,753 (91.6)	159 (5.8)
	Yes	623 (8.6)	50 (8.0)	370 (8.7)	34 (9.2)	253 (8.4)	16 (6.3)
	No	6,666 (91.7)	376 (5.6)	3,919 (92.0)	249 (6.4)	2,747 (91.4)	127 (4.6)

**Variables of interest**		**Births irrespective of Covid-19 peak period**		**Births in Covid-19 peak period**		**Births in Covid-19 non-peak period**	
		**All births**	**Stillbirths**	**All births**	**Stillbirths**	**All births**	**Stillbirths**
		***N* = 7,270 (% of N)**	***N* = 501 (% of births)**	***N* = 4,263 (% of N)**	***N* = 326 (% of births)**	***N* = 3,007 (% of N)**	***N* = 175 (% of births)**
**Delivery was referred to another health care provider**[b]	Yes	602 (8.3)	124 (20.6)	343 (8.0)	76 (22.2)	259 (8.6)	48 (18.5)
**Place of delivery**	Public facility	3,707 (51.0)	128 (3.5)	2,110 (49.5)	81 (3.8)	1,597 (53.1)	47 (2.9)
	Private facility	1,873 (25.8)	243 (13.0)	1,121 (26.3)	155 (13.8)	752 (25.0)	88 (11.7)
	Home	1,573 (21.6)	116 (7.4)	964 (22.6)	79 (8.2)	609 (20.3)	37 (6.1)
	On route to facility	116 (1.6)	13 (11.2)	67 (1.6)	10 (14.9)	49 (1.6)	3 (6.1)
**Spontaneous labour**[b]	No/Don't know	777 (10.7)	98 (12.6)	472 (11.1)	72 (15.3)	305 (10.2)	26 (8.5)
	Yes	6,491 (89.3)	402 (6.2)	3,790 (88.9)	253 (6.7)	2,701 (89.8)	149 (5.5)
**Foul smelling discharge**[b]	No/Don't know	7,001 (96.3)	461 (6.6)	4,116 (96.6)	296 (7.2)	2,885 (96.0)	165 (5.7)
	Yes	267 (3.7)	39 (14.6)	146 (3.4)	29 (19.9)	121 (4.0)	10 (8.3)
**Vaginal delivery**[b]	No/Don't know	951 (13.1)	95 (10.0)	543 (12.7)	59 (10.9)	408 (13.6)	36 (8.8)
	Yes	6,317 (86.9)	405 (6.4)	3,719 (87.3)	266 (7.2)	2,598 (86.4)	139 (5.4)
**Push/forceful pull done during delivery by health care provider**[b]	No/Don't know	6,741 (92.8)	374 (5.6)	3,959 (92.9)	236 (6.0)	2,782 (92.5)	138 (5.0)
	Yes	527 (7.3)	126 (23.9)	303 (7.1)	89 (29.4)	224 (7.5)	37 (16.5)
**Entangled cord around baby's neck**[b]	No/Don't know	7,126 (98.1)	492 (6.9)	4,182 (98.1)	319 (7.6)	2,944 (97.9)	173 (5.6)
	Yes	142 (1.9)	8 (5.6)	80 (1.9)	6 (7.5)	62 (2.1)	2 (3.2)
**Breech position of the baby**[d]	No/Don't know	6,893 (94.9)	428 (6.2)	4,030 (94.6)	281 (7.0)	2,863 (95.3)	147 (5.1)
	Yes	373 (5.1)	72 (19.3)	231 (5.4)	44 (19.1)	142 (4.7)	28 (19.7)
**Number of months in last trimester of pregnancy during the Covid-19 peak period**	0	1,172 (16.1)	61 (5.2)	NA	NA	1,172 (39.0)	61 (5.2)
	1	1,829 (25.2)	103 (5.6)	1,230 (28.9)	68 (5.5)	599 (19.9)	35 (5.8)
	2	2,406 (33.1)	182 (7.6)	1,170 (27.5)	103 (8.8)	1,236 (41.1)	79 (6.4)
	3	1,863 (25.6)	155 (8.3)	1,863 (43.7)	155 (8.3)	NA	NA
**Covid-19 peak period birth**	No	3,007 (41.4)	175 (5.8)	NA	NA	NA	NA
	Yes	4,263 (58.6)	326 (7.7)	NA	NA	NA	NA

[a]Data missing for 6 births; [b]Data missing for 2 births; [c]Data missing for 1 birth; [d]Data missing for 4 births; NA= Not applicable

Supplementary Table 4-. Distribution of select variables by the number of months in the last trimester of pregnancy during the Covid-19 peak period.

Variables of interest		Number of months in the last trimester of pregnancy that were in the Covid-19 peak period			
		**0 month N=1,172 (% of N)**	**1 month N=1,829 (% of N)**	**2 months N=2,406 (% of N)**	**3 months N=1,863 (% of N)**
**Gestational age at the time of delivery[a]**	7 months	29 (2.5)	46 (2.5)	84 (3.5)	64 (3.4)
	8 months	358 (30.6)	581 (31.8)	767 (31.9)	587 (31.5)
	>8 months	785 (67.0)	1,202 (65.7)	1,555 (64.6)	1,212 (65.1)
**Delivery was deferred[b,c]**	Yes	106 (9.1)	154 (8.4)	203 (8.4)	160 (8.6)
**Mother was referred for delivery to another health care provider[b,d]**	Yes	102 (8.7)	142 (7.8)	198 (8.2)	160 (8.6)
**Place of delivery[e,f]**	Public	599 (51.1)	990 (54.1)	1,198 (49.8)	920 (49.4)
	Private	314 (26.8)	435 (23.8)	634 (26.4)	490 (26.3)
	Home	259 (22.1)	404 (22.1)	574 (23.9)	452 (24.3)

[a]Chi-square test p-value=0.359 for gestation period at the time of delivery
[b]Data missing for 2 births
[c]Chi-square test p-value = 0.929 for delivery was deferred
[d]Chi-square test p-value =0.759 for mother was referred for delivery to another health care provider
[e]Data missing for 1 birth; [f]Chi-square test p-value=0.065 for place of delivery

Supplementary Table 5. Results of sequential multiple logistic regression models 1-4 for association of stillbirth with socio-demographic, maternal, pregnancy, labour, and delivery related risk factors in the Indian state of Bihar for all births.

Risk factor	Risk factor category	Adjusted odds ratio for stillbirth (95% confidence interval)			
		Model 1*	Model 2*	Model 3*	Model 4*
**Place of residence**	Rural	1.05 (0.82-1.34)	1.02 (0.79-1.32)	1.01 (0.79-1.29)	0.95 (0.74-1.22)
	Urban	1.00	1.00	1.00	1.00
**Sex of the baby**	Boy	1.34 (1.11-1.61)	1.34 (1.11-1.61)	1.33 (1.10-1.61)	1.30 (1.07-1.58)
	Girl	1.00	1.00	1.00	1.00
**Wealth index quartile**	I (lowest)	1.06 (0.81-1.38)‡			
	II	1.06 (0.81-1.38)‡			
	III	0.89 (0.68-1.17)‡			
	IV (highest)	1.00			
**Maternal age**	15-19 years		0.98 (0.59-1.62)	0.88 (0.52-1.49)	0.92 (0.54-1.57)
	20-24 years		0.77 (0.53-1.14)	0.73 (0.49-1.10)	0.74 (0.49-1.11)
	25-29 years		0.76 (0.52-1.12)	0.73 (0.49-1.09)	0.74 (0.49-1.11)
	30-34 years		0.94 (0.62-1.44)	0.94 (0.61-1.46)	0.97 (0.62-1.50)
	>=35 years		1.00	1.00	1.00
**Solid cooking fuel use**	Yes		1.03 (0.83-1.28)‡		
	No		1.00		
**First born**	Yes		1.28 (1.01-1.63)	1.41 (1.09-1.82)	1.15 (0.89-1.50)‡
	No		1.00	1.00	1.00
**Previous history of stillbirth**	Yes		2.33 (1.71-3.18)	2.14 (1.54-2.97)	2.05 (1.47-2.87)
	No		1.00	1.00	1.00
**Previous history of miscarriage**	Yes		1.17 (0.90-1.50)‡		
	No		1.00		
**Maternal antenatal care visit during pregnancy**	No			1.52 (1.02-2.26)	1.76 (1.17-2.63)
	Yes			1.00	1.00
**Mother received 2 tetanus toxoid injections during pregnancy**	Yes			1.15 (0.93-1.43)	1.23 (0.99-1.53)
	No			1.00	1.00
**Mother consumed Iron Folic Acid tablets during pregnancy**	Yes			1.26 (0.99-1.59)	1.24 (0.97-1.57)
	No			1.00	1.00
**Pregnancy with multiple foetuses**	Yes			1.29 (0.81-2.04)‡	
	No			1.00	
**Maternal hypertension in the last trimester of pregnancy**	Yes			1.43 (0.89-2.32)	1.19 (0.73-1.95)‡
	No			1.00	1.00
	Yes			1.05 (0.31-3.57)‡	

Risk factor	Risk factor category	Adjusted odds ratio for stillbirth (95% confidence interval)			
		Model 1*	Model 2*	Model 3*	Model 4*
**Mother had malaria in the last trimester of pregnancy**	No			1.00	
**Mother diagnosed with syphilis during this pregnancy**	Yes			0.76 (0.45-1.30)‡	
	No			1.00	
**Mother had fever in the last 3 months of pregnancy**	Yes			1.31 (0.94-1.83)	1.29 (0.93-1.80)
	No			1.00	1.00
**Mother had convulsions in the last 3 months of pregnancy**	Yes			1.09 (0.83-1.44)‡	
	No			1.00	
**Mother informed baby was not growing adequately inside the womb**	Yes			1.10 (0.83-1.47)‡	
	No			1.00	
**Number of months in last trimester of pregnancy during the Covid-19 peak period**	0			1.00	1.00
	1			1.05 (0.75-1.46)	1.04 (0.74-1.45)
	2			1.39 (1.02-1.89)	1.44 (1.05-1.96)
	3			1.51 (1.10-2.06)	1.53 (1.11-2.11)
**Gestation age**	7 months			9.45 (6.87-13.00)	9.28 (6.72-12.81)
	>7-8 months			1.54 (1.26-1.89)	1.57 (1.28-1.93)
	>8 months			1.00	1.00
**Deferred delivery**	Yes				1.22 (0.88-1.68)‡
	No				1.00
**Delivery was referred to another health care provider**	Yes				4.15 (3.25-5.30)
	No				1.00
**Spontaneous labour**	Yes				0.58 (0.45-0.75)
	No				1.00

* Sex of the baby and place of residence considered a priori in all models regardless of significance
‡P≥0.2, and hence excluded from the next model

Dandona R, *et al. BMJ Glob Health* 2023; 8:e013021. doi: 10.1136/bmjgh-2023-013021

BMJ Publishing Group Limited (BMJ) disclaims all liability and responsibility arising from any reliance placed on this supplemental material which has been supplied by the author(s)

Supplementary Table 6. Results of sequential multiple logistic regression models 1-4 for association of stillbirth with socio-demographic, maternal, pregnancy, labour, and delivery related risk factors in the Indian state of Bihar for births during the Covid-19 peak period. NA denotes not applicable.

Risk factor	Risk factor category	Adjusted odds ratio for stillbirth during the Covid-19 peak period (95% confidence interval)			
		Model 1*	Model 2*	Model 3*	Model 4*
**Place of residence**	Rural	1.09 (0.81-1.46)	1.09 (0.80-1.49)	1.08 (0.80-1.45)	1.00 (0.74-1.35)
	Urban	1.00	1.00	1.00	1.00
**Sex of the baby**	Boy	1.23 (0.98-1.54)	1.24 (0.98-1.56)	1.24 (0.98-1.57)	1.23 (0.97-1.57)
	Girl	1.00	1.00	1.00	1.00
**Wealth index quartile**	I (lowest)	0.98 (0.71-1.35)[‡]			
	II	0.89 (0.64-1.25)[‡]			
	III	0.79 (0.57-1.10)[‡]			
	IV (highest)	1.00			
**Maternal age**	15-19 years		1.08 (0.57-2.05)[‡]		
	20-24 years		0.82 (0.49-1.36)[‡]		
	25-29 years		0.83 (0.50-1.37)[‡]		
	30-34 years		0.97 (0.56-1.70)[‡]		
	>=35 years		1.00		
**Solid cooking fuel use**	Yes		1.01 (0.77-1.33)[‡]		
	No		1.00		
**First born**	Yes		1.51 (1.13-2.02)	1.65 (1.26-2.17)	1.27 (0.97-1.66)
	No		1.00	1.00	1.00
**Previous history of stillbirth**	Yes		2.55 (1.73-3.77)	2.37 (1.57-3.58)	2.31 (1.52-3.50)
	No		1.00	1.00	1.00
**Previous history of miscarriage**	Yes		1.36 (1.00-1.85)	1.36 (0.99-1.87)	1.21 (0.88-1.68)[‡]
	No		1.00		
**Maternal antenatal care visit during pregnancy**	No			1.21 (0.72-2.02)[‡]	
	Yes			1.00	
**Mother received 2 tetanus toxoid injections during pregnancy**	Yes			1.17 (0.89-1.53)[‡]	
	No			1.00	
**Mother consumed Iron Folic Acid tablets during pregnancy**	Yes			1.40 (1.05-1.87)	1.53 (1.17-2.00)
	No			1.00	1.00
**Pregnancy with multiple foetuses**	Yes			1.25 (0.70-2.21)[‡]	
	No			1.00	
**Maternal hypertension in the last trimester of pregnancy**	Yes			1.46 (0.80-2.66)[‡]	
	No			1.00	
	Yes			0.51 (0.06-4.14)[‡]	

Dandona R, *et al. BMJ Glob Health* 2023; 8:e013021. doi: 10.1136/bmjgh-2023-013021

Risk factor	Risk factor category	Adjusted odds ratio for stillbirth during the Covid-19 peak period (95% confidence interval)			
		Model 1*	Model 2*	Model 3*	Model 4*
**Mother had malaria in the last trimester of pregnancy**	No			1.00	
**Mother diagnosed with syphilis during this pregnancy**	Yes			0.83 (0.43-1.59)‡	
	No			1.00	
**Mother had fever in the last 3 months of pregnancy**	Yes			1.76 (1.20-2.58)	1.75 (1.21-2.54)
	No			1.00	1.00
**Mother had convulsions in the last 3 months of pregnancy**	Yes			0.85 (0.58-1.23)‡	
	No			1.00	
**Mother informed baby was not growing adequately inside the womb**	Yes			1.09 (0.77-1.56)‡	
	No			1.00	
**Number of months in last trimester of pregnancy during the Covid-19 peak period**	0			NA	NA
	1			1.00	1.00
	2			1.58 (1.14-2.19)	1.62 (1.16-2.26)
	3			1.48 (1.09-2.00)	1.48 (1.09-2.02)
**Gestation age**	7 months			9.24 (6.21-13.77)	9.39 (6.31-13.96)
	>7-8 months			1.45 (1.12-1.88)	1.49 (1.15-1.94)
	>8 months			1.00	1.00
**Deferred delivery**	Yes				1.22 (0.82-1.81)‡
	No				1.00
**Delivery was referred to another health care provider**	Yes				3.68 (2.69-5.04)
	No				1.00
**Spontaneous labour**	Yes				0.53 (0.39-0.72)
	No				1.00

* Sex of the baby and place of residence considered a priori in all models regardless of significance
‡P≥0.2, and hence excluded from the next model

Supplementary Table 7. Results of sequential multiple logistic regression models 1-4 for association of stillbirth with socio-demographic, maternal, pregnancy, labour, and delivery related risk factors in the Indian state of Bihar for births during the Covid-19 non-peak period. NA denotes not applicable.

Risk factor	Risk factor category	Adjusted odds ratio for stillbirth during the Covid-19 peak period (95% confidence interval)			
		Model 1*	Model 2*	Model 3*	Model 4*
**Place of residence**	Rural	0.93 (0.60-1.44)	0.89 (0.56-1.42)	0.87 (0.55-1.38)	0.89 (0.56-1.42)
	Urban	1.00	1.00	1.00	1.00
**Sex of the baby**	Boy	1.57 (1.15-2.16)	1.57 (1.14-2.15)	1.58 (1.14-2.19)	1.49 (1.07-2.07)
	Girl	1.00	1.00	1.00	1.00
**Wealth index quartile**	I (lowest)	1.25 (0.77-2.01)‡	1.23 (0.73-2.08)‡	1.10 (0.66-1.82)‡	1.21 (0.72-2.01)
	II	1.49 (0.94-2.35)	1.47 (0.91-2.38)	1.54 (0.96-2.46)	1.67 (1.03-2.70)
	III	1.17 (0.73-1.89)‡	1.17 (0.72-1.91)‡	1.16 (0.71-1.89)‡	1.19 (0.72-1.96)
	IV (highest)	1.00	1.00	1.00	1.00
**Maternal age**	15-19 years		0.87 (0.37-2.07)‡		
	20-24 years		0.72 (0.39-1.34)‡		
	25-29 years		0.68 (0.37-1.26)‡		
	30-34 years		0.94 (0.49-1.82)‡		
	>=35 years		1.00		
**Solid cooking fuel use**	Yes		1.14 (0.77-1.68)‡		
	No		1.00		
**First born**	Yes		0.90 (0.58-1.41)‡		
	No		1.00		
**Previous history of stillbirth**	Yes		2.01 (1.20-3.39)	1.91 (1.12-3.26)	1.87 (1.08-3.23)
	No		1.00	1.00	1.00
**Previous history of miscarriage**	Yes		0.88 (0.55-1.39)‡		
	No		1.00		
**Maternal antenatal care visit during pregnancy**	No			2.33 (1.22-4.45)	2.99 (1.71-5.25)
	Yes			1.00	1.00
**Mother received 2 tetanus toxoid injections during pregnancy**	Yes			1.09 (0.77-1.53)‡	
	No			1.00	
**Mother consumed Iron Folic Acid tablets during pregnancy**	Yes			1.07 (0.71-1.60)‡	
	No			1.00	
**Pregnancy with multiple foetuses**	Yes			1.45 (0.65-3.25)‡	
	No			1.00	
**Maternal hypertension in the last trimester of pregnancy**	Yes			1.25 (0.54-2.89)‡	
	No			1.00	
	Yes			2.00 (0.43-9.40)‡	

Dandona R, *et al. BMJ Glob Health* 2023; 8:e013021. doi: 10.1136/bmjgh-2023-013021

Risk factor	Risk factor category	Adjusted odds ratio for stillbirth during the Covid-19 peak period (95% confidence interval)			
		Model 1*	Model 2*	Model 3*	Model 4*
**Mother had malaria in the last trimester of pregnancy**	No			1.00	
**Mother diagnosed with syphilis during this pregnancy**	Yes			0.73 (0.28-1.89)‡	
	No			1.00	
**Mother had fever in the last 3 months of pregnancy**	Yes			0.58 (0.27-1.25)	0.57 (0.27-1.22)
	No			1.00	1.00
**Mother had convulsions in the last 3 months of pregnancy**	Yes			1.56 (1.02-2.39)	1.41 (0.92-2.16)
	No			1.00	1.00
**Mother informed baby was not growing adequately inside the womb**	Yes			1.17 (0.71-1.94)‡	
	No			1.00	
**Number of months in last trimester of pregnancy during the Covid-19 peak period**	0			1.00	
	1			1.09 (0.70-1.69)‡	
	2			1.15 (0.81-1.64) ‡	
	3			NA	
**Gestation age**	7 months			10.41 (5.99-18.08)	10.35 (5.94-18.03)
	>7-8 months			1.71 (1.22-2.40)	1.76 (1.25-2.48)
	>8 months			1.00	1.00
**Deferred delivery**	Yes				1.06 (0.60-1.85)‡
	No				1.00
**Delivery was referred to another health care provider**	Yes				4.70 (3.20-6.92)
	No				1.00
**Spontaneous labour**	Yes				0.78 (0.49-1.25)‡
	No				1.00

* Sex of the baby and place of residence considered a priori in all models regardless of significance
‡P≥0.2, and hence excluded from the next model

Dandona R, *et al. BMJ Glob Health* 2023; 8:e013021. doi: 10.1136/bmjgh-2023-013021

Supplementary Figure 1. Classification process used to identify antepartum and intrapartum deaths.[18]

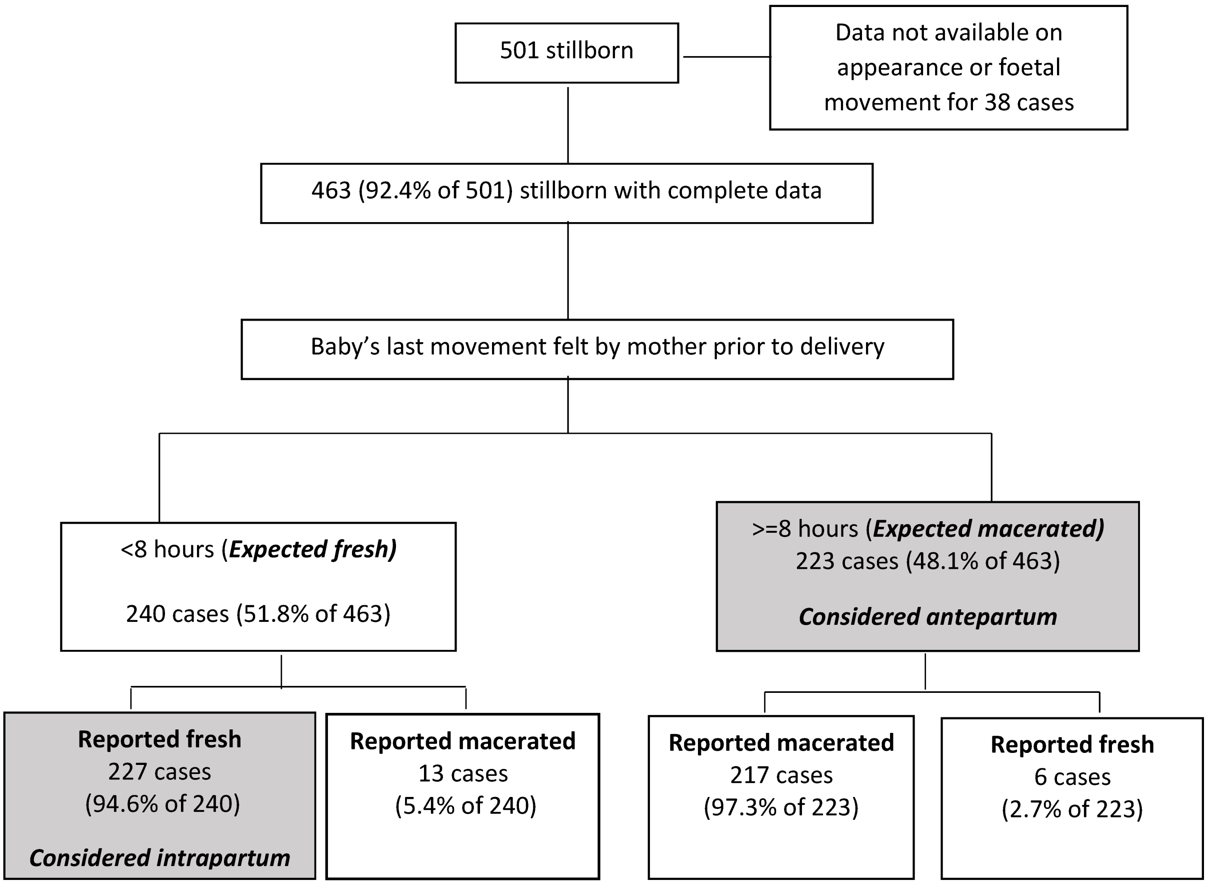

Dandona R, *et al. BMJ Glob Health* 2023; 8:e013021. doi: 10.1136/bmjgh-2023-013021

Supplementary Figure 2. **Distribution of stillbirths among women based on the number of months in the last trimester of pregnancy that were in the Covid-19 peak period, 2020-21.**

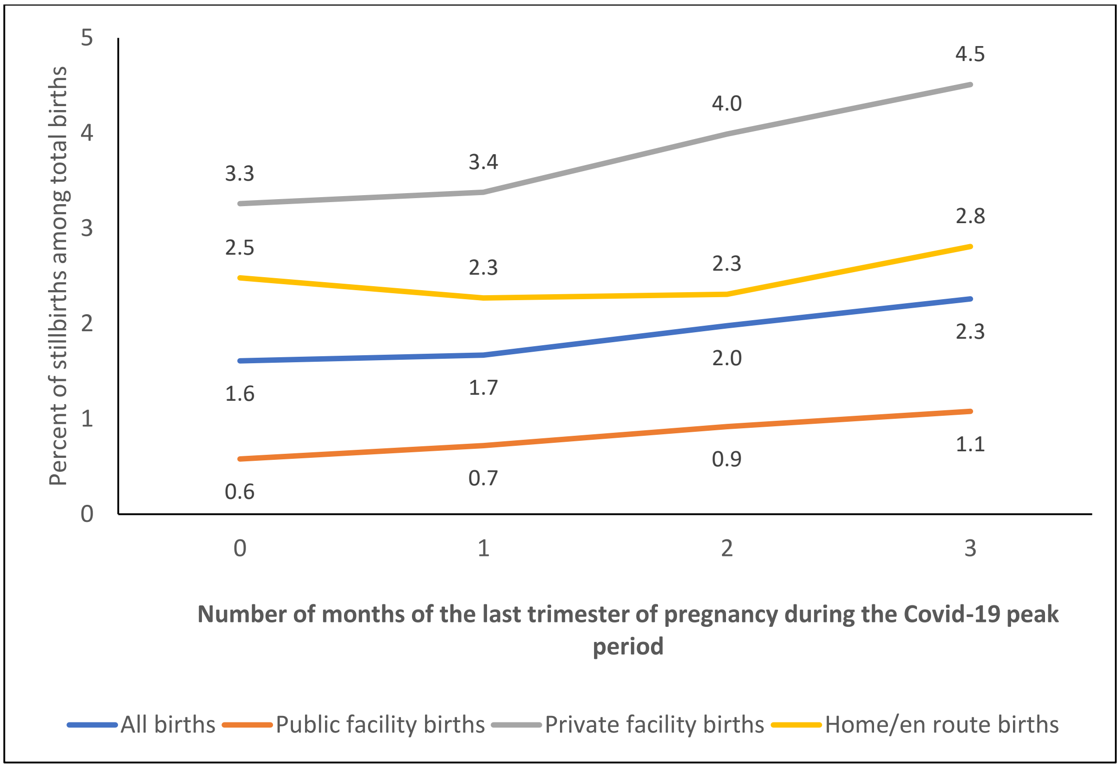

Supplementary Figure 3. Distribution of the number of antenatal care (ANC) visits among women based on the number of months in the last trimester of pregnancy that were in the Covid-19 peak period.

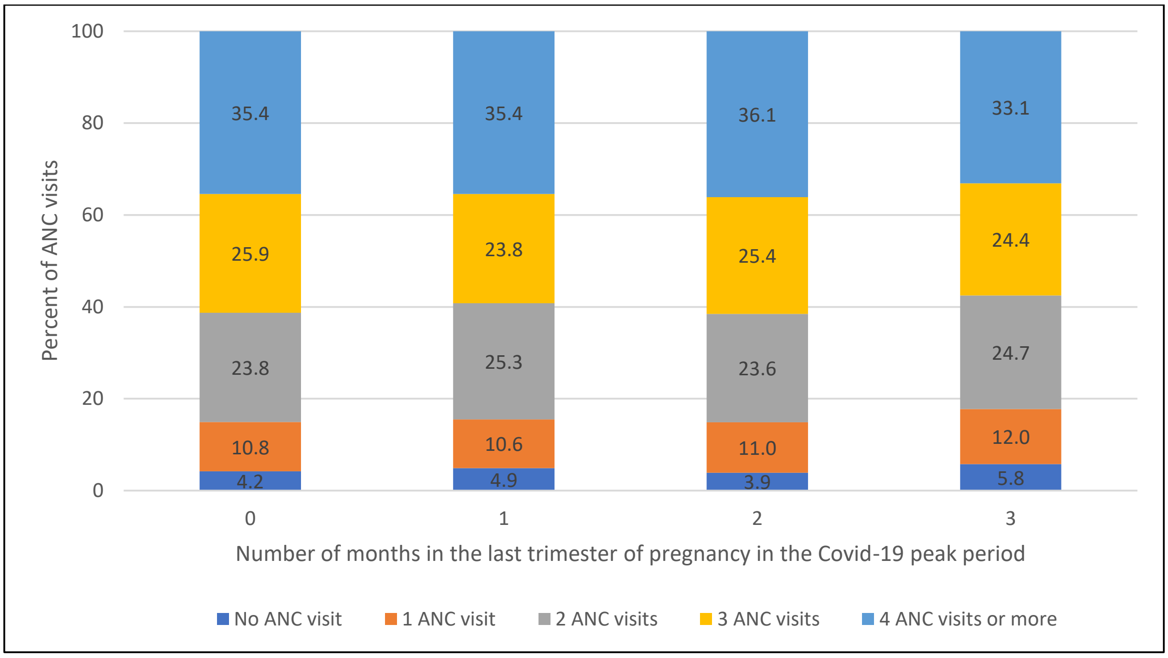

Supplement: Supplementary data [file bmjgh-2023-013021supp001.pdf]
